# Community-level social capital and polypharmacy among public assistance recipients in Japan: A multilevel cross-sectional study

**DOI:** 10.1016/j.ssmph.2025.101788

**Published:** 2025-03-20

**Authors:** Masayuki Kasahara, Haruna Kawachi, Keiko Ueno, Shiho Kino, Naoki Kondo, Shunya Ikeda, Daisuke Nishioka

**Affiliations:** a Department of Medical and Biopharmaceutical Sciences, Graduate School of Pharmacy, International University of Health and Welfare, 4-1-26 Akasaka, Minato-ku, Tokyo, Japan; b Department of Medical Statistics, Medical Research & Development Center, Osaka Medical and Pharmaceutical University, Daigakumachi 2-7, Takatsuki-shi, Osaka, Japan; c Department of Social Epidemiology, Graduate School of Medicine and School of Public Health, Kyoto University, Floor 2, Science Frontier Laboratory, Yoshida-konoe-cho, Sakyo-ku, Kyoto-shi, Kyoto, Japan; d Department of Preventive Oral Health Care Sciences, Graduate School of Medical and Dental Sciences, Institute of Science Tokyo, 1-5-45 Yushima, Bunkyo-ku, Tokyo, Japan; e Department of Social Impact Assessment, Graduate School of Medicine and School of Public Health, Kyoto University, Floor 2, Science Frontier Laboratory, Yoshida-Konoe-cho, Sakyo-ku, Kyoto-shi, Kyoto, Japan

## Abstract

• Polypharmacy is common among low socioeconomic public assistance recipients.
• 69.5 % of adults on public assistance had polypharmacy with ≥6 oral medications.
• Higher civic participation was associated with lower excessive polypharmacy prevalence.
• Higher social cohesion was associated with higher excessive polypharmacy prevalence.
• Polypharmacy should be addressed based on civic participation and social cohesion.

## 1 Introduction

Polypharmacy, commonly defined as the concomitant use of multiple medications, is a growing health concern and a worldwide challenge (World Health Organization, 2019, World Health Organization, 2016). Polypharmacy prevalence among older adults is reported to be 30–40 % of the population across developed countries (Ministry of Health, Labour and Welfare, 2018; Midão et al., 2018; Young et al., 2021). People with polypharmacy experience an increased risk of drug-drug and drug-disease interactions, adverse drug events, hospitalization, and mortality (Chang et al., 2020; Huang et al., 2021; Toh et al., 2023). Individual biopsychological factors such as multimorbidity, aging, and mental illness require multiple medications (Menditto et al., 2019; Nicholson et al., 2024), resulting in medication-related harm (Calderón-Larrañaga et al., 2012). Individual socioeconomic factors are also important predictors of polypharmacy (Iqbal et al., 2023). Individuals with low socioeconomic status, including income (Feng et al., 2018), level of education (Castioni et al., 2017), wealth (Slater et al., 2018), social class (Thomas et al., 1999), and social isolation (Svensson et al., 2023).

To effectively tackle the global issue of polypharmacy, it is crucial to address polypharmacy risks through a socio-ecological approach. In the context of health promotion strategies, McLeroy's socio-ecological model provides a comprehensive framework for intervention (McLeroy et al., 1988). This model categorizes strategies into individual, interpersonal, institutional, community, and public policy levels. Previous research has extensively examined individual and interpersonal-level factors associated with polypharmacy from a bio-psycho-social perspective. Institutional interventions, such as healthcare system-based programs aimed at optimizing medication use, have been widely implemented (Daunt et al., 2023). Additionally, government-led policies have been introduced to regulate polypharmacy (Ministry of Health, Labour and Welfare, 2018; Scottish Government Polypharmacy Model of Care Group, 2018). Despite these efforts, polypharmacy remains disproportionately prevalent among socioeconomically disadvantaged populations (Iqbal et al., 2023; Kim et al., 2014). This phenomenon may be attributed to cognitive and behavioral characteristics specific to these populations (Mani et al., 2013), which hinder optimal medication management. Prior studies have highlighted that behavioral change is particularly challenging for socioeconomically disadvantaged individuals due to a complex interplay of socioeconomic factors (Nagelhout et al., 2017). To effectively address this challenge, community-level interventions have been increasingly recognized as essential (O'Mara-Eves et al., 2015). However, research on the impact of community-level factors on polypharmacy prevalence remains limited. Given that social environments play a crucial role in shaping healthcare utilization and medication adherence (Kawachi & Berkman, 2014; Kim & Kawachi, 2017), it is imperative to investigate whether community-level social factors influence the risk of polypharmacy. The World Health Organization (WHO) has also encouraged countries to safeguard their populations from the negative health impacts of polypharmacy, considering socioeconomic contexts (World Health Organization, 2019, World Health Organization, 2002).

Social capital, defined as the health-enhancing resources accessible to an individual through their network or group membership (Berkman et al., 2014; Kawachi et al., 2008), is one of the potential measures that can address the negative impact of social health determinant factors on polypharmacy. For people with low socioeconomic status, community-level social capital has a greater positive impact on health outcomes (Uphoff et al., 2013). Greater social capital may mitigate the risk of prevalence among residents; however, addressing issues of polypharmacy among socially disadvantaged populations may need further consideration of the dark side of social capital. For them, fostering the social capital may lead to undesirable health outcomes, suffering its stress through exclusionary pathways (e.g., not belonging, peer pressure) (Amemiya et al., 2019; Haseda et al., 2018; Portes, 1998).

Socioeconomically disadvantaged populations can access welfare programs designed to alleviate financial hardship and support health protection (World Health Organization, 2012). In Japan, the governmental welfare program “public assistance” (seikatsu-hogo) provides comprehensive financial coverage for housing, medical care, and long-term care costs, along with a minimum livelihood income for individuals living in poverty (Sakamoto et al., 2018). Individuals below the poverty line and without any assets can apply for this program, with local governments conducting rigorous means-testing to determine eligibility. Approximately 1.6 % of Japan's population in fiscal year 2021 uses this program (Ministry of Internal Affairs and Communications, n.d.). Japan's universal healthcare system allows free choice of medical providers to medical services without gatekeeping, and public assistance recipients have their basic living and healthcare needs financially secured. Consequently, they constitute a unique population with minimal opportunity costs and virtually no financial barriers to healthcare utilization. This distinct institutional framework provides a valuable opportunity to examine the potential impact of community-level social capital on polypharmacy prevalence among socioeconomically disadvantaged populations, isolating the effects of the social capital from financial constraints on healthcare access.

Therefore, in this study, we aimed to investigate the association between community-level social capital and the status of polypharmacy among socially disadvantaged populations using the data of public assistance recipients in Japan.

## 2 Materials and methods

### 2.1 Study design

This was a cross-sectional study.

### 2.2 Study population

**Fig. 1:**
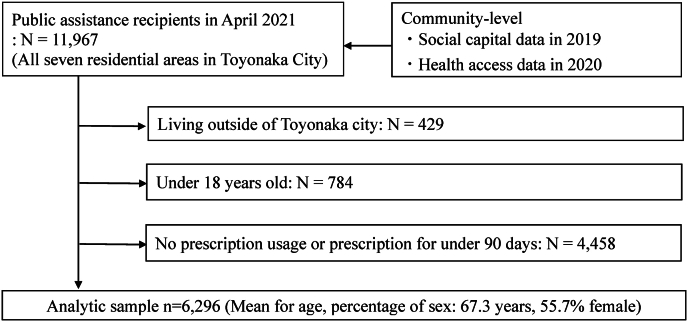
Participants flow for the analytic sample.

Fig. 1 shows the participants’ flowchart for the analytic sample. This study included all 11,967 individuals who had received public assistance in Toyonaka City, Osaka, Japan, in April 2021. We excluded 429 individuals living outside of the city, 784 aged <18 years, and 4458 with no prescription usage or prescription for <90 days. The analytic samples included 6296 individuals aged ≥18 years with the prescribed medications for ≥90 days, consistent with several previous studies (Bjerrum et al., 1997; Guthrie et al., 2015; Ministry of Health, Labour and Welfare, 2021b; Ishizaki et al., 2020; Nishtala & Salahudeen, 2015).

Toyonaka City is an urban city neighboring the north of central Osaka with population of approximately 0.4 million people, and 1.1 habitable population density (in Japan: 1.0). The classification system consists of three major tiers, with Toyonaka City being in the highest tier. Notably, about 60 % of public assistance recipients in Japan reside in first-tier areas (Portal Site of Official Statistics of Japan, n.d.), indicating a concentration of welfare beneficiaries in regions with higher living costs. A percentage of public assistance recipients of 2.4 % (average in Japan: 1.6 %), while the distribution of recipients’ demographic characteristics is consistent with previous studies (Nishioka et al., 2022; Sengoku et al., 2022).

### 2.3 Data sources

#### 2.3.1 Individual-level data

We used the public assistance database of the municipal welfare office. This dataset, with no missing data, included the sociodemographic data in April 2021, such as age, sex, household composition, nationality, employment status, income (including working income, pensions, and disability pensions), long-term care status, and health checkups. We used the recipient's administrative medical claims data from April 2021 to March 2022. The claims data included the recipient's use of medical care each month, such as medical institution codes, diagnosis codes, drug codes of prescribed medications, and prescription duration in days (Ministry of Health, Labour and Welfare, n.d.-d). Each individual was merged with the public assistance database and medical claims data using unique identification codes.

#### 2.3.2 Community-level data

We used the recipient's regional information comprising healthcare access and community-level social capital data. To obtain healthcare access data from the municipal welfare office, our dataset included the number of medical institutions in the elementary school district in 2020. Community-level social capital data comprised the 2019 Japan Gerontological Evaluation Study (JAGES) data in units of everyday living areas (e.g., junior high school districts). The elementary and junior high school districts (Toyonaka City, 2021, Toyonaka City, 2022, Toyonaka City, 2023, 2022, 2023) unite where individuals travel outside on foot or bicycle (Iwai-Saito et al., 2021; Ministry of Education, Culture, Sports, Science, and Technology, n.d.). The JAGES survey is a triennial nationwide investigation of the social determinants of healthy aging in community-dwelling older adults. The JAGES 2019 survey included 278,000 individuals in 39 municipalities, and 196,000 valid responses (70.5 % response rate). In Toyonaka City, a total of 3379 individuals responded out of the 6150 sampled participants (54.9 % response rate). Each recipient's area was merged with community-level healthcare access and social capital data using district codes.

### 2.4 Measurement and variables

#### 2.4.1 Outcome variables

Polypharmacy status, used as an outcome, was measured using prescription duration in days and drug codes of the 12 digits listed in the National Health Insurance Prices Standard codes (Ministry of Health, Labour and Welfare, n.d.-a). First, the oral prescription medications were identified using the first 5–7 digits of the drug code. Second, the prescription of at least one drug of the same efficacy classification for ≥90 days was determined using the first four digits of the drug code and prescription duration in days (Ishizaki et al., 2020). Third, after excluding duplicate components from different medical institutions, the number of prescribed drugs was tabulated for each individual.

We defined the following three groups of polypharmacy status based on the Japanese guidelines (Ministry of Health, Labour and Welfare, 2018) and Medication Safety in Polypharmacy reported by WHO (World Health Organization, 2019): the reference group (1–5 medications); the polypharmacy group (6–9 medications); and the excessive polypharmacy group (≥10 medications).

#### 2.4.2 Explanatory variables

The three scores of community-level social capital, calculated from the JAGES 2019, were used as the explanatory variables. These scores, based on indicators developed and validated by Saito et al. (Saito et al., 2017), comprised civic participation (i.e., the behavioral manifestations of network connections or civic engagement), social cohesion (i.e., the subjective attitudes, such as trust, norms of reciprocity, and attachment within the community), and reciprocity (i.e., the exchange of individual social support within the community); subsequently, they were calculated as percentages for seven units of everyday living areas of the recipients.

#### 2.4.3 Covariates

##### 2.4.3.1 Individual-level covariates

We controlled for individual-level variables, including sociodemographic data such as age (18–29/30–39/40–49/50–59/60–64/65–74/75–84/≥85 years), sex (female/male), household composition (living together/living alone), employment status (unemployed/employed), disability certificate (no/yes), and long-term care status (none/support needed/long-term care needed). Long-term care status was determined using seven levels of care, which are assessed through the nationally standardized Long-Term Care Insurance system in Japan as follows: support needed, levels 1–2 (e.g., support-1 and -2, which are a condition requiring assistance in daily living); and long-term care needed, levels 3–7 (e.g., long-term care-1–5, which are a condition requiring constant care owing to bedridden or dementia) (Ministry of Health, Labour and Welfare, n.d.-c; Tsutsui & Muramatsu, 2005). Eligibility for long-term care is for all individuals aged ≥65 years and those aged 40–64 years with specific diseases (Ministry of Health, Labour and Welfare, n.d.-b). Additionally, we used the Charlson Comorbidity Index (CCI) (continuous) from diagnosis codes of the claims data. The CCI predicts the mortality rate for chronic diseases (Charlson, Pompei, Ales, & MacKenzie, 1987) and is frequently used as a variable to represent the patient's statuses of multimorbidity, which are associated with polypharmacy (Arabyat et al., 2021; Tefera et al., 2020). As additional covariates, we included health checkups (no/yes) and the number of different medical institutions visited in the fiscal year (continuous) based on medical institution codes from the claims data. People with multiple medical institutions have a higher polypharmacy prevalence (Suzuki et al., 2020).

##### 2.4.3.2 Community-level covariates

The variable included the number of medical institutions (continuous) in the elementary school district as the geographical accessibility to healthcare.

### 2.5 Statistical analyses

The participants’ characteristics were described based on the prevalence risk of polypharmacy stratified accordingly to all (≥18) and age groups (18–39/40–64/≥65) (analysis-1). In analysis-1, the median and interquartile range were calculated for continuous variables and number (N) and percentages (%) for categorical variables. Next, the multilevel multinomial logistic regression model was used to investigate the association between community-level social capital scores and polypharmacy status among public assistance recipients to evaluate the potential for community-level interventions. The odds ratio (OR) and 95 % confidence interval (CI) of each variable were calculated (analysis-2). As a subgroup, we performed analysis-2 stratified based on age groups (18–39/40–64/≥65) to consider the availability of health resources. All statistical analyses were performed using STATA MP 17 (STATA Corp. LLC, College Station, TX, USA).

## 3 Results

This study included 6296 individuals with an average age of 67.3 years, of whom 55.7 % were female. The number of prescription drugs and each variable were stratified according to age group, as presented in Tables 1, S1, S2, and S3. Among all (≥18) and age groups (18–39/40–64/≥65), the prevalence of polypharmacy (including excessive polypharmacy) was 4377 (69.5 %), 111 (44.9 %), 1106 (69.2 %), and 3160 (71.0 %) in individuals aged ≥18, 18–39, 40–64, and ≥65 years, respectively. In all age groups, an increase in age, higher CCI scores, and more frequent visits to medical institutions were associated with a higher prevalence of polypharmacy and excessive polypharmacy. For civic participation scores among individuals aged 18–39 and 40–64 years, first quartile range values were lower in the excessive polypharmacy group than in the reference group (1–5 medications). Among all (≥18) and age groups (18–39/40–64/≥65), the values for social cohesion and reciprocity scores were higher in the excessive polypharmacy group than in the reference group (1–5 medications).

**Table 1:** Characteristics of participants with polypharmacy among public assistance recipients aged ≥18 years.

	≥18 years old			
	Total	Number of oral medicines		
		Reference (1–5 medications)	Polypharmacy (6–9 medications)	Excessive polypharmacy (≥10 medications)
	N = 6296	n = 1919	n = 2508	n = 1869
		(30.5 % for N)	(39.8 % for N)	(29.7 % for N)
**Community variables (median, IQR)**				
Daily living areas				
Civic participation		8.5 (7.3–10.6)	8.5 (7.3–10.4)	8.5 (7.3–10.6)
Social cohesion		58.2 (56.7–61.4)	58.2 (56.7–61.4)	58.2 (58.2–61.4)
Reciprocity		89.9 (88.8–91.3)	89.9 (88.8–91.3)	89.9 (89.9–91.3)
Number of medical institutions		15.0 (7.0–23.0)	16.0 (7.0–23.0)	13.0 (7.0–23.0)
**Individual variables**				
Age				
18–29	96	76 (79.2)	16 (16.7)	4 (4.1)
30–39	151	60 (39.7)	68 (45.0)	23 (15.3)
40–49	326	115 (35.3)	110 (33.7)	101 (31.0)
50–59	834	265 (31.8)	293 (35.1)	276 (33.1)
60–64	437	111 (25.4)	158 (36.2)	168 (38.4)
65–74	1118	343 (30.7)	470 (42.0)	305 (27.3)
75–84	2079	629 (30.3)	853 (41.0)	597 (28.7)
≥85	1255	320 (25.5)	540 (43.0)	395 (31.5)
Sex				
Female	3510	1039 (29.6)	1396 (39.8)	1075 (30.6)
Male	2786	880 (31.6)	1112 (39.9)	794 (28.5)
Household composition				
Living together	1603	549 (34.3)	590 (36.8)	464 (28.9)
Living alone	4693	1370 (29.2)	1918 (40.9)	1405 (29.9)
Employment status				
Unemployed	5861	1709 (29.2)	2359 (40.2)	1793 (30.6)
Employed	435	210 (48.3)	149 (34.2)	76 (17.5)
Disability certificate				
No	4756	1576 (33.2)	1914 (40.2)	1266 (26.6)
Yes	1540	343 (22.3)	594 (38.6)	603 (39.1)
Long-term care status				
None	3885	1368 (35.2)	1495 (38.5)	1022 (26.3)
Support needed	952	227 (23.8)	396 (41.6)	329 (34.6)
Long-term care needed	1459	324 (22.2)	617 (42.3)	518 (35.5)
CCI (median, IQR)		1.0 (0.0–2.0)	2.0 (1.0–3.0)	2.0 (1.0–4.0)
Health checkups				
No	5955	1793 (30.1)	2404 (40.4)	1758 (29.5)
Yes	341	126 (36.9)	104 (30.5)	111 (32.6)
Number of different medical institutions visited/year (median, IQR)		4.0 (2.0–5.0)	4.0 (3.0–6.0)	6.0 (4.0–8.0)

Note: IQR, Interquartile range; CCI, Charlson Comorbidity Index.

Table 2 presents the adjusted ORs and 95 % CIs for the association between each social capital indicator and polypharmacy prevalence among public assistance recipients aged ≥18 years. Higher civic participation was associated with lower excessive polypharmacy prevalence (adjusted OR: 0.89, 95 % CI: 0.83–0.96), whereas higher social cohesion was associated with higher excessive polypharmacy prevalence (adjusted OR: 1.06, 95 % CI: 1.01–1.10). However, reciprocity was not significantly associated with polypharmacy prevalence overall. In subgroup analyses, similar trends were observed among adults aged 40 years and older (Tables S5 and S6). However, in younger adults (18–39 years), there was an inconsistent pattern, where higher social cohesion tended to be associated with lower excessive polypharmacy prevalence (adjusted OR: 0.88, 95 % CI: 0.64–1.20), while higher reciprocity was linked to higher excessive polypharmacy prevalence (adjusted OR: 2.17, 95 % CI: 0.97–4.87), although not statistically significant.

**Table 2:** Community-level social capital scores associated with polypharmacy among public assistance recipients aged ≥18 years.

	≥18 years old N = 6296				
	Reference (1–5 medications)	Crude		Adjusted	
		Polypharmacy (6–9 medications)	Excessive polypharmacy (≥10 medications)	Polypharmacy (6–9 medications)	Excessive polypharmacy (≥10 medications)
		OR (95 % CI)			
**Community variables (Daily living areas)**					
Civic participation		0.95 (0.90, 1.01)	**0.92** (0.86, 0.98)	0.96 (0.90, 1.02)	**0.89** (0.83, 0.96)
Social cohesion		1.02 (0.98, 1.05)	**1.05** (1.02, 1.09)	1.00 (0.97, 1.04)	**1.06** (1.01, 1.10)
Reciprocity		0.97 (0.90, 1.05)	0.98 (0.91, 1.07)	1.00 (0.92, 1.09)	0.99 (0.89, 1.09)

Note: Results in bold font are statistically significant; OR, Odds ratio; CI, Confidence interval; Multinomial logistic regression models adjusted for covariates including individual-level (age, sex, household composition, employment status, disability certificate, long-term care status, Charlson Comorbidity Index, health checkups, and number of different medical institutions visited/year) and community-level (number of medical institutions) variables.

## 4 Discussion

Our study found that a higher community-level civic participation score showed a lower excessive polypharmacy prevalence among public assistance recipients. Additionally, in all age groups (≥18/18–39/40–64/≥65), the association between civic participation and excessive polypharmacy was similar. Conversely, a higher social cohesion score showed a higher excessive polypharmacy prevalence. Reciprocity showed a tendency toward a higher polypharmacy prevalence only among younger adult recipients.

The findings of this study indicate that promoting civic participation among older adults in a community may help in addressing polypharmacy among public assistance recipients. Several studies have reported that greater community-level civic participation is beneficial for health-related outcomes, including hypertension (Nakagomi et al., 2019) and depressive symptoms (Yamaguchi et al., 2019), as well as disability (Fujihara et al., 2019). Community-level civic participation may positively impact health outcomes via positive social interaction, self-efficacy, and health behavior pathways (Berkman et al., 2000). Therefore, a positive aspect of community-level civic participation, which mitigates individuals with social isolation (Berkman et al., 2014), potentially reduces adverse health outcomes, including polypharmacy. The finding, which is observed across different age groups, indicates that fostering community-level civic participation among older adults might lower the prevalence of polypharmacy among younger recipients. Previous studies found that civic participation among older people and young children in Japan can influence and increase civic participation across other generations, including people of different ages (Murayama et al., 2012; Murayama et al., 2019). Additionally, encouraging social interaction across generations in community-dwelling adults is associated with improved mental health among young and older adults (Nemoto et al., 2018; Nemoto et al., 2022).

Community-building support across generations has been promoted through a framework in a community-based inclusive society. Part of this framework also includes a community-based integrated care system targeting the older population (Ministry of Health, Labour and Welfare, 2021a; Otaga, 2024). In the community-based integrated care system, Japanese guidelines for the appropriate prescription for older people indicate that the prevalence of polypharmacy is mitigated through collaboration among healthcare professionals (e.g., prescribing physicians, pharmacists, and other members of the multidisciplinary team) (Ministry of Health, Labour and Welfare, 2018). However, when public assistance recipients experience the prevalence of disease approximately 10 years earlier than the general public insurance population (Sengoku et al., 2022), polypharmacy measures for older adults in community-based integrated care may be an oversight among younger recipients. Promoting civic participation may also help to mitigate the polypharmacy prevalence among younger public assistance recipients who are excluded from eligible populations in community-based integrated care.

Strong social cohesion within a community may have complex effects on a higher polypharmacy prevalence, particularly among marginalized populations, including public assistance recipients. Several studies have reported that strong social cohesion is associated with improved access to healthcare (Calciolari & Luini, 2023; Kim & Kawachi, 2017; Mizuochi, 2016). It is also associated with a lower prevalence of depressive symptoms among the general older population; however, in socially vulnerable populations with low socioeconomic status, no lower impact prevalence of depressive symptoms is observed than that in affluent people (Haseda et al., 2018). Socially vulnerable populations within a greater community social cohesion are susceptible to the psychosocial stress of stigma through the experience of social exclusion (Pickett & Wilkinson, 2008), thereby increasing the risk of polypharmacy by promoting healthcare access.

The lack of a significant association between reciprocity and polypharmacy may suggest that the benefits of community-level reciprocity are not equally distributed among socially disadvantaged populations, such as public assistance recipients. Prior research indicated that reciprocity tends to benefit individuals with greater socioeconomic resources, whereas disadvantaged groups may face exclusion or limited access to mutual aid (Portes, 1998; Uphoff et al., 2013). Additionally, social stigma may prevent disadvantaged populations from fully integrating into reciprocal networks, reducing potential health benefits (Haseda et al., 2018). Among younger adults, the observed trend suggesting a positive association between reciprocity and excessive polypharmacy may be due to community welfare structures in Japan. Older adults, who are more likely to engage in voluntary activities, often provide support to younger, socially disadvantaged individuals through meal assistance for children and parents, and employment support (Certified NPO National Children's Cafeteria Support Center—Musubie—, 2024; Toyonaka city, n.d.). This might lead to improved healthcare access and increased polypharmacy prevalence in younger adults, though further research is needed to confirm this relationship.

This study had several strengths. First, to the best of our knowledge, this is the first study to examine the possible contextual relationship between community-level social capital and polypharmacy among public assistance recipients. Second, we could examine the association between residents’ polypharmacy and their residential community-level social capital using linkage data from the public assistance database and the community-level social capital data of JAGES.

In contrast, we acknowledge some limitations. First, in medical claims data from all public assistance recipients, this study may underestimate the polypharmacy prevalence because of potentially having polypharmacy among reference groups (1–5 medications). Some recipients receive the medications in combination with other welfare programs (e.g., welfare support for severe mental disabilities (Ministry of Health, Labour and Welfare, n.d.) or intractable diseases (Japan Intractable Diseases Information Center, n.d.)). For example, along with the support for severe mental disorders, public assistance recipients apply for other welfare support to treat mental diseases, whereas non-applicable conditions (e.g., diabetes, hypertension, and asthma) are covered by public assistance welfare programs. Second, using the data from a single municipality limits generalizability provided that several materials suggest applicability with municipalities with varying urbanization levels. Though our findings may be broadly applicable to urban settings where most recipients reside given that the characteristics of public assistance recipients in this study resemble the research reported in previous studies in Japan (Nishioka et al., 2022; Sengoku et al., 2022), we acknowledge that Toyonaka City may have a relatively lower social capital score compared to other municipalities in Japan (Takeuchi et al., 2022), and the response rate in the JAGES survey was lower than the national average. Additional validation across multiple municipalities is necessary to fully establish the external validity of community-level social capital effects. Third, the validity of the community-level unit remains unclear. Our study used a community-level unit because this is a school district where recipients can travel outside on foot or bicycle (Iwai-Saito et al., 2021; Ministry of Education, Culture, Sports, Science, and Technology, n.d.). However, this district may not be the area where recipients actually live. Therefore, future research should include the geographic unit indicators reflecting the residential area of recipients. Fourth, as a measure of multimorbidity, the CCI may not sufficiently adjust for confounding factors in the association between community-level social capital and polypharmacy prevalence. It is frequently difficult for the CCI to express the number of conditions owing to the mortality risk index (Fortin et al., 2005). Hence, there are potentially residual confounding factors. Fifth, the multilevel analysis of this study could not consider individual-level social capital, as it only accessed community-level data for older adults. A previous study shows that older adults with lower individual-level social cohesion in highly cohesive communities take longer to improve their functional disability than those with higher cohesion. This suggests that older adults experience social exclusion and alienation (Amemiya et al., 2019), which should be examined in future studies. However, due to survey participation difficulties in impoverished populations, data on individual-level social capital proved highly challenging (Emery et al., 2023). Sixth, since we could only use the public assistance database, the findings of this study cannot be generalized to impoverished populations who were ineligible for public assistance programs. Finally, as a cross-sectional study, our research undetermined causal pathways. Polypharmacy prescribed for ≥90 days was calculated in 1 year, including baseline. Future studies should follow up with individuals without polypharmacy at baseline to determine the incidence of polypharmacy.

## 5 Conclusions

Our findings suggest a contextual relationship between community-level social capital and polypharmacy among public assistance recipients. Specifically, higher civic participation among older adults was associated with lower polypharmacy prevalence, whereas higher social cohesion was linked to an increased risk of excessive polypharmacy. Additionally, community-level reciprocity was slightly associated with polypharmacy only among younger adult recipients. Addressing polypharmacy among public assistance recipients requires a community-based approach. In addition to promoting civic participation, it is crucial to develop social norms that prevent the exclusion of socially vulnerable populations and mitigate the negative effects of strong social cohesion. Further research using data from multiple municipalities, longitudinal designs, and individual-level social capital measures is necessary to better understand the mechanisms underlying these associations.

## CRediT authorship contribution statement

**Masayuki Kasahara:** Writing – original draft, Validation, Methodology, Formal analysis, Data curation, Conceptualization. **Haruna Kawachi:** Writing – review & editing, Validation, Methodology, Investigation, Data curation. **Keiko Ueno:** Writing – review & editing, Validation. **Shiho Kino:** Writing – review & editing, Validation. **Naoki Kondo:** Writing – review & editing, Validation, Resources. **Shunya Ikeda:** Writing – review & editing, Supervision, Methodology. **Daisuke Nishioka:** Writing – review & editing, Validation, Supervision, Project administration, Investigation, Funding acquisition, Data curation.

## Ethical statement

The Ethics Committee of Osaka Medical and Pharmaceutical University (No. 2022-089) and the International University of Health and Welfare (No. 23-Ig-129) approved the study protocol.

## Funding

This work was supported by the Japan Society for the Promotion of Science KAKENHI grant (22K17404) and the Ministry of Health Labour and Welfare (Health Labor Sciences Special Research Grant (23CA2001), Health Labor Sciences Research on Policy Planning and Evaluation (24AA2004).

## Declaration of interest statement

The authors declare no conflicts of interest statement associated with this research.

## Data Availability

The authors do not have permission to share data.
