# Supplementary material for: Community-level social capital and polypharmacy among public assistance recipients in Japan: A multilevel cross-sectional study
Source: SSM Popul Health. 2025 Mar 20;30:101788. doi: 10.1016/j.ssmph.2025.101788 (PMC11984595; doi:10.1016/j.ssmph.2025.101788)
Supplement: Multimedia component 1 [file mmc1.docx]

| Table S1. Among individuals aged 18–39 years, characteristics of participants with polypharmacy among public assistance recipients | | | | | |
| --- | --- | --- | --- | --- | --- |
|  |  | 18–39 years old | | | |
|  |  | Total | Number of oral medicines | | |
|  |  |  | Reference  (1–5 medications) | Polypharmacy (6–9 medications) | Excessive polypharmacy (≥10 medications) |
|  |  | N=247 | n=136 | n=84 | n=27 |
|  |  |  | (55.1% for N) | (34.0% for N) | (10.9% for N) |
| **Community variables (median, IQR)** | | | |  |  |
| Daily living areas |  |  |  |  |  |
| Civic participation | |  | 9.4 (8.5–10.6) | 9.4 (8.5–10.6) | 9.4 (7.3–10.6) |
| Social cohesion |  |  | 59.9 (56.7–62.2) | 59.9 (58.2–61.4) | 59.9 (58.2–65.2) |
| Reciprocity |  |  | 90.3 (88.8–91.3) | 90.7 (89.9–91.3) | 90.3 (89.9–91.3) |
| Number of medical institutions | | | 13.0 (6.0–17.0) | 15.5 (7.0–32.0) | 16.0 (7.0–32.0) |
| **Individual variables** | | |  |  |  |
| Age |  |  |  |  |  |
| 18–29 |  | 96 | 76 (79.2) | 16 (16.7) | 4 (4.1) |
| 30–39 |  | 151 | 60 (39.7) | 68 (45.0) | 23 (15.3) |
| Sex |  |  |  |  |  |
| Female | | 143 | 74 (51.7) | 53 (37.1) | 16 (11.2) |
| Male |  | 104 | 62 (59.6) | 31 (29.8) | 11 (10.6) |
| Household composition | |  |  |  |  |
| Living together | | 156 | 91 (58.3) | 51 (32.7) | 14 (9.0) |
| Living alone |  | 91 | 45 (49.5) | 33 (36.2) | 13 (14.3) |
| Employment status | |  |  |  |  |
| Unemployed | | 218 | 120 (55.0) | 75 (34.4) | 23 (10.6) |
| Employed | | 29 | 16 (55.2) | 9 (31.0) | 4 (13.8) |
| Disability certificate | |  |  |  |  |
| No |  | 173 | 102 (59.0) | 58 (33.5) | 13 (7.5) |
| Yes |  | 74 | 34 (46.0) | 26 (35.1) | 14 (18.9) |
| CCI (median, IQR) | |  | 0.0 (0.0–1.0) | 1.0 (0.0–1.0) | 1.0 (1.0–2.0) |
| Health checkups |  |  |  |  |  |
| No |  | 237 | 129 (54.4) | 83 (35.0) | 25 (10.6) |
| Yes |  | 10 | 7 (70.0) | 1 (10.0) | 2 (20.0) |
| Number of different medical institutions visited/year (median, IQR) | | | 4.0 (3.0–6.0) | 5.0 (3.0–8.0) | 7.0 (5.0–10.0) |
| Note: IQR, Interquartile range; CCI, Charlson Comorbidity Index. | | | | | |

| Table S2. Among individuals aged 40–64 years, characteristics of participants with polypharmacy among public assistance recipients | | | | | |
| --- | --- | --- | --- | --- | --- |
|  |  | 40–64 years old | | | |
|  |  | Total | Number of oral medicines | | |
|  |  |  | Reference  (1–5 medications) | Polypharmacy (6–9 medications) | Excessive polypharmacy (≥10 medications) |
|  |  | N=1597 | n=491 | n=561 | n=545 |
|  |  |  | (30.8% for N) | (35.1% for N) | (34.1% for N) |
| **Community variables (median, IQR)** | | | |  |  |
| Daily living areas |  |  |  |  |  |
| Civic participation | |  | 8.5 (8.5–10.6) | 8.5 (8.5–10.6) | 8.5 (7.3–10.6) |
| Social cohesion |  |  | 58.2 (56.7–61.4) | 58.2 (56.7–61.4) | 58.2 (58.2–61.4) |
| Reciprocity |  |  | 89.9 (88.8–91.3) | 89.9 (88.8–91.3) | 89.9 (89.9–91.3) |
| Number of medical institutions | | | 16.0 (7.0–23.0) | 16.0 (7.0–26.0) | 16.0 (7.0–31.0) |
| **Individual variables** | | |  |  |  |
| Age |  |  |  |  |  |
| 40–49 |  | 326 | 115 (35.3) | 110 (33.7) | 101 (31.0) |
| 50–59 |  | 834 | 265 (31.8) | 293 (35.1) | 276 (33.1) |
| 60–64 |  | 437 | 111 (25.4) | 158 (36.2) | 168 (38.4) |
| Sex |  |  |  |  |  |
| Female | | 889 | 259 (29.2) | 307 (34.5) | 323 (36.3) |
| Male |  | 708 | 232 (32.8) | 254 (35.9) | 222 (31.3) |
| Household composition | |  |  |  |  |
| Living together | | 542 | 188 (34.7) | 174 (32.1) | 180 (33.2) |
| Living alone |  | 1,055 | 303 (28.7) | 387 (36.7) | 365 (34.6) |
| Employment status | |  |  |  |  |
| Unemployed | | 1,342 | 372 (27.7) | 480 (35.8) | 490 (36.5) |
| Employed | | 255 | 119 (46.6) | 81 (31.8) | 55 (21.6) |
| Disability certificate | |  |  |  |  |
| No |  | 1,029 | 352 (34.2) | 376 (36.5) | 301 (29.3) |
| Yes |  | 568 | 139 (24.5) | 185 (32.6) | 244 (42.9) |
| Long-term care status | |  |  |  |  |
| None |  | 1,508 | 473 (31.4) | 525 (34.8) | 510 (33.8) |
| Support needed | | 28 | 4 (14.3) | 11 (39.3) | 13 (46.4) |
| Long-term care needed | | 61 | 14 (22.9) | 25 (41.0) | 22 (36.1) |
| CCI (median, IQR) | |  | 1.0 (0.0–2.0) | 1.0 (0.0–2.0) | 2.0 (1.0–3.0) |
| Health checkups |  |  |  |  |  |
| No |  | 1,490 | 446 (29.9) | 531 (35.7) | 513 (34.4) |
| Yes |  | 107 | 45 (42.1) | 30 (28.0) | 32 (29.9) |
| Number of different medical institutions visited/year (median, IQR) | | | 4.0 (3.0–6.0) | 4.0 (3.0–7.0) | 6.0 (4.0–9.0) |
| Note: IQR, Interquartile range; CCI, Charlson Comorbidity Index. | | | | | |

| Table S3. Among individuals aged ≥65 years, characteristics of participants with polypharmacy among public assistance recipients | | | | | |
| --- | --- | --- | --- | --- | --- |
|  |  | ≥65 years old | | | |
|  |  | Total | Number of oral medicines | | |
|  |  |  | Reference  (1–5 medications) | Polypharmacy (6–9 medications) | Excessive polypharmacy (≥10 medications) |
|  |  | N=4452 | n=1292 | n=1863 | n=1297 |
|  |  |  | (29.0% for N) | (41.9% for N) | (29.1% for N) |
| **Community variables (median, IQR)** | | | |  |  |
| Daily living areas |  |  |  |  |  |
| Civic participation | |  | 8.5 (7.3–10.4) | 8.5 (7.3–10.4) | 8.5 (7.3–10.4) |
| Social cohesion |  |  | 58.2 (56.7–61.4) | 58.2 (56.7–61.4) | 58.2 (58.2–61.4) |
| Reciprocity |  |  | 89.9 (88.8–91.3) | 89.9 (88.8–91.3) | 89.9 (89.9–91.3) |
| Number of medical institutions | | | 13.0 (7.0–23.0) | 13.0 (7.0–23.0) | 13.0 (7.0–23.0) |
| **Individual variables** | | |  |  |  |
| Age |  |  |  |  |  |
| 65–74 |  | 1,118 | 343 (30.7) | 470 (42.0) | 305 (27.3) |
| 75–84 |  | 2,079 | 629 (30.3) | 853 (41.0) | 597 (28.7) |
| ≥85 |  | 1,255 | 320 (25.5) | 540 (43.0) | 395 (31.5) |
| Sex |  |  |  |  |  |
| Female | | 2,478 | 706 (28.5) | 1,036 (41.8) | 736 (29.7) |
| Male |  | 1,974 | 586 (29.7) | 827 (41.9) | 561 (28.4) |
| Household composition | |  |  |  |  |
| Living together | | 905 | 270 (29.8) | 365 (40.3) | 270 (29.9) |
| Living alone |  | 3,547 | 1,022 (28.8) | 1,498 (42.2) | 1,027 (29.0) |
| Employment status | |  |  |  |  |
| Unemployed | | 4,301 | 1,217 (28.3) | 1,804 (41.9) | 1,280 (29.8) |
| Employed | | 151 | 75 (49.6) | 59 (39.1) | 17 (11.3) |
| Disability certificate | |  |  |  |  |
| No |  | 3,554 | 1,122 (31.6) | 1,480 (41.6) | 952 (26.8) |
| Yes |  | 898 | 170 (18.9) | 383 (42.7) | 345 (38.4) |
| Long-term care status | |  |  |  |  |
| None |  | 2,130 | 759 (35.6) | 886 (41.6) | 485 (22.8) |
| Support needed | | 924 | 223 (24.1) | 385 (41.7) | 316 (34.2) |
| Long-term care needed | | 1,398 | 310 (22.2) | 592 (42.3) | 496 (35.5) |
| CCI (median, IQR) | |  | 1.0 (1.0–3.0) | 2.0 (1.0–3.0) | 3.0 (2.0–4.0) |
| Health checkups |  |  |  |  |  |
| No |  | 4,228 | 1,218 (28.8) | 1,790 (42.3) | 1,220 (28.9) |
| Yes |  | 224 | 74 (33.0) | 73 (32.6) | 77 (34.4) |
| Number of different medical institutions visited/year (median, IQR) | | | 4.0 (2.0–5.0) | 4.0 (3.0–6.0) | 6.0 (4.0–8.0) |
| Note: IQR, Interquartile range; CCI, Charlson Comorbidity Index. | | | | | |

| Table S4. Among individuals aged 18–39 years, community-level social capital scores are associated with polypharmacy among public assistance recipients | | | | | | |
| --- | --- | --- | --- | --- | --- | --- |
|  |  | 18–39 years old N = 247 | | | | |
|  | Reference  (1–5 medications) | Crude | |  | Adjusted | |
|  |  | Polypharmacy (6–9 medications) | Excessive polypharmacy (≥10 medications) |  | Polypharmacy (6–9 medications) | Excessive polypharmacy (≥10 medications) |
|  |  | OR (95% CI) | | | | |
| **Community variables (Daily living areas)** | | | |  |  |  |
| Civic participation | | 1.03 (0.78, 1.36) | 0.81 (0.53, 1.26) |  | 1.09 (0.78, 1.52) | 0.74 (0.39, 1.37) |
| Social cohesion |  | 1.01 (0.88, 1.15) | 1.07 (0.87, 1.31) |  | 0.96 (0.81, 1.13) | 0.88 (0.64, 1.20) |
| Reciprocity |  | 0.97 (0.69, 1.36) | 1.11 (0.68, 1.82) |  | 1.20 (0.80, 1.80) | 2.17 (0.97, 4.87) |
| Note: Results in bold font are statistically significant; OR, Odds ratio; CI, Confidence interval; Multinomial logistic regression models adjusted for covariates including individual-level (age, sex, household composition, employment status, disability certificate, Charlson Comorbidity Index, health checkups, and number of different medical institutions visited/year) and community-level (number of medical institutions) variables. | | | | | | |

| Table S5. Among individuals aged 40–64 years, community-level social capital scores are associated with polypharmacy among public assistance recipients | | | | | | |
| --- | --- | --- | --- | --- | --- | --- |
|  |  | 40–64 years old N = 1,597 | | | | |
|  | Reference  (1–5 medications) | Crude | |  | Adjusted | |
|  |  | Polypharmacy (6–9 medications) | Excessive polypharmacy (≥10 medications) |  | Polypharmacy (6–9 medications) | Excessive polypharmacy (≥10 medications) |
|  |  | OR (95% CI) | | | | |
| **Community variables (Daily living areas)** | |  |  |  |  |  |
| Civic participation |  | 0.99 (0.87, 1.12) | **0.88** (0.77, 0.99) |  | 0.96 (0.84, 1.09) | **0.81** (0.71, 0.93) |
| Social cohesion |  | 1.00 (0.94, 1.07) | **1.09** (1.03, 1.16) |  | 0.99 (0.92, 1.06) | **1.08** (1.00^＊^, 1.16) |
| Reciprocity |  | 0.96 (0.83, 1.12) | 0.95 (0.81, 1.11) |  | 1.03 (0.87, 1.21) | 1.02 (0.84, 1.23) |
| Note: Results in bold font are statistically significant; OR, Odds ratio; CI, Confidence interval; Multinomial logistic regression models adjusted for covariates including individual-level (age, sex, household composition, employment status, disability certificate, long-term care status, Charlson Comorbidity Index, health checkups, and number of different medical institutions visited/year) and community-level (number of medical institution) variables; Asterisk is greater than 1.00. | | | | | | |

| Table S6. Among individuals aged ≥65 years, community-level social capital scores associated with polypharmacy among public assistance recipients | | | | | | |
| --- | --- | --- | --- | --- | --- | --- |
|  |  | ≥65 years old N = 4,452 | | | | |
|  | Reference  (1–5 medications) | Crude | |  | Adjusted | |
|  |  | Polypharmacy (6–9 medications) | Excessive polypharmacy (≥10 medications) |  | Polypharmacy (6–9 medications) | Excessive polypharmacy (≥10 medications) |
|  |  | OR (95% CI) | | | | |
| **Community variables (Daily living areas)** | | | |  |  |  |
| Civic participation |  | 0.95 (0.89, 1.03) | 0.95 (0.88, 1.03) |  | 0.94 (0.87, 1.02) | 0.93 (0.85, 1.02) |
| Social cohesion | | 1.02 (0.98, 1.06) | 1.03 (0.99, 1.08) |  | 1.02 (0.97, 1.06) | 1.05 (1.00, 1.11) |
| Reciprocity |  | 0.97 (0.89, 1.06) | 1.00 (0.90, 1.10) |  | 0.97 (0.88, 1.08) | 0.96 (0.85, 1.08) |
| Note: Results in bold font are statistically significant; OR, Odds ratio; CI, Confidence interval; Multinomial logistic regression models adjusted for covariates including individual-level (age, sex, household composition, employment status, disability certificate, long-term care status, Charlson Comorbidity Index, health checkups, and number of different medical institutions visited/year) and community-level (number of medical institution) variables. | | | | | | |
